# The relationship between colleagues’ social support and burnout: a scoping review of quantitative research

**DOI:** 10.3389/fpsyg.2026.1858058

**Published:** 2026-10-06

**Authors:** Élodie Latour, Mathias Rossi, François Lauzier-Jobin, Roxane Rousseau, Sophie Meunier

**Affiliations:** 1 Department of Psychology, Université du Québec à Montréal, Montreal, QC, Canada; 2 School of Nursing, Faculty of Medicine and Health Sciences, Université de Sherbrooke, Montreal, QC, Canada

**Keywords:** burnout, colleagues, emotional exhaustion, scoping review, social support

## Abstract

**Introduction:**

Social support, particularly from colleagues, is recognized as an important protective factor against burnout. However, the literature on this association is fragmented, making it difficult to draw any conclusions and orient future research. To fill this gap, this study seeks to map the extent, range and nature of the quantitative scientific literature examining the relationship between colleagues’ social support and burnout.

**Methods:**

A scoping review was performed on nine databases, with a total of 154 studies included.

**Results:**

Most studies were cross-sectional, were conducted in Europe and North America and examined workers from the health and social services sector. A wide variety of questionnaires were used to measure colleagues’ social support, while the majority of studies used the Maslach Burnout Inventory to measure burnout. Although there has been a significant increase in studies examining the relationship between colleagues’ social support and burnout over the years, most such studies were conducted with homogeneous samples. Very few studies (7.8%) discuss aspects of diversity among the participants (e.g., gender, ethnicity). Studies also lack consistency in terms of the measurements used to assess colleagues’ social support and thus the obtained results.

**Discussion:**

Further studies that use longitudinal designs, apply comprehensive and multidimensional measures of social support and focus on participants with diverse attributes (e.g., non-heterosexual participants, participants living with a mental illness) are needed to provide a more nuanced understanding of the role of colleagues’ social support in burnout.

## 1 Introduction

Psychological health problems at work affect a large number of individuals and represent a major issue for organizations. About 15% of working-age adults suffer from a mental disorder (World Health Organization, 2024) and one in five workers experiences high or very high levels of stress at work (Statistics Canada, 2023). Over a prolonged period, this stress can lead to significant consequences for workers, including burnout, which is defined as a syndrome resulting from chronic workplace stress that has not been successfully regulated (Leiter and Maslach, 2024). Social support, particularly from colleagues, appears to be a central factor in playing a protective role against burnout (Avanzi et al., 2018; Chiaburu and Harrison, 2008; Inoue et al., 2022; Kilroy et al., 2022). Indeed, colleagues provide daily support and mutual understanding of the workplace environment, which may be especially relevant for coping with emotional strain and stress at work (Inoue et al., 2022). Given that the effects of social support may vary depending on its source, it is important to examine social support from colleagues independently of other sources, such as supervisor or family members (Jolly et al., 2021). However, the literature on social support at work is extensive and disparate (Jolly et al., 2021). Studies are usually insufficiently precise in their measurement of social support and do not make distinctions in terms of its sources (e.g., colleagues, supervisors) or functions (e.g., emotional support, instrumental support; Jolly et al., 2021), making it difficult to draw clear conclusions and to guide future research regarding the association between colleagues’ social support and burnout. To address this shortcoming, the present study sets out to conduct a scoping review to map the characteristics of the studies investigating the association between colleagues’ social support and burnout, including how these constructs have been operationalized, and examined, in order to identify gaps and guide future research and intervention in a more systematic way.

### 1.1 Burnout

The phenomenon of burnout was introduced in the 1970s by Freudenberger (1974) and Maslach (1976). Though it was first observed among people in helping professions, it is now recognized as a phenomenon that may occur in any sector (Schaufeli and Buunk, 2003). Burnout is usually characterized by three main dimensions: (1) emotional exhaustion: a feeling of exhaustion or lack of energy; (2) depersonalization or cynicism: increased mental distance with others or with one’s work, and feelings of negativism or cynicism linked to one’s work; and (3) reduced professional effectiveness: reduced perception of accomplishment (Leiter and Maslach, 2024). This conceptualization and its associated questionnaire, the Maslach Burnout Inventory, or MBI (Maslach and Jackson, 1981), remains the most well-known and widely used in the scientific literature. However, it has been criticized over the years for lacking a theoretical basis and failing to provide an overall burnout score (Shirom, 2005). Indeed, each dimension of the MBI has been associated with different antecedents and consequences, and it is recommended that they be considered separately (Lee and Ashforth, 1996). In response to these critiques, other conceptualizations and questionnaires of burnout have emerged, such as the Copenhagen Burnout Inventory (CBI; Kristensen et al., 2005), which measures burnout in three different domains (personal burnout, work-related burnout and client-related burnout), or the Oldenburg Burnout Inventory (OLBI; Demerouti et al., 2003), which measures two dimensions of burnout (exhaustion and disengagement). However, the MBI or one of its subscales is still used in 90% of burnout studies (Villacura-Herrera et al., 2025).

Burnout has been associated with various consequences that are physical (e.g., cardiovascular diseases, hypercholesterolemia, type 2 diabetes), psychological (e.g., insomnia, depressive symptoms, symptoms of psychological ill-health) and occupational (e.g., absenteeism, presenteeism and job dissatisfaction; Salvagioni et al., 2017). However, some variables have been identified as protective factors against burnout and its negative effects. Among these variables, social support from colleagues has been largely examined in a variety of contexts and is therefore particularly relevant to consider (Avanzi et al., 2018; Chiaburu and Harrison, 2008; Inoue et al., 2022; Kilroy et al., 2022).

### 1.2 Social support from colleagues

Social support refers to “psychological or material resources that are provided to a focal individual by partners in some form of social relationship” (Jolly et al., 2021, p. 229). In the workplace, House (1981) identified four main functions of social support: (1) emotional support (empathy, listening and caring); (2) instrumental support (concrete and tangible help); (3) informational support (advice, useful information, suggestions); and (4) appraisal support (providing information to help a person evaluate themselves or their situation). Social support can come from a wide variety of sources, such as colleagues, supervisors and family members (Jolly et al., 2021).

Social support from colleagues seems particularly important when it comes to overcoming psychological health difficulties, such as burnout. For example, the study by Inoue et al. (2022), conducted among employees from 13 companies in Japan, shows that colleagues’ social support (and not that of the supervisor) was associated with a decrease in psychological distress. In the same vein, the study by Macias-Velasquez et al. (2021), conducted among middle and senior managers from the Mexican manufacturing industry, indicates that colleagues’ social support differed from that of supervisors in relation to the different dimensions of burnout: while colleagues’ support was negatively associated with emotional exhaustion, supervisors’ support was negatively associated with cynicism. These variations concerning the effect of social support from colleagues or supervisors could be explained by the different relational dynamics involved. These two sources of support vary in terms of their proximity to employees and how frequently they interact with them, as well as in the hierarchical relationship they have with them (Inoue et al., 2022). This could lead employees to appreciate more emotional support from colleagues and more work-related support from a supervisor. Overall, social support from colleagues and social support from supervisors both serve as important defenses against burnout, but in different ways, which underscores the importance of studying them separately.

### 1.3 The association between colleagues’ social support (CSS) and burnout

Several theoretical models offer explanations for the association between CSS and burnout. First, according to the Job Demands-Resources Model (JD-R; Bakker and Demerouti, 2007), CSS could be considered a job resource, defined as “physical, psychological, social, or organizational aspects of the job that help achieve work goals, reduce job demands and their associated physiological and psychological costs, and stimulate personal growth, learning, and development” (Bakker and Demerouti, 2007, p. 312). In the JD-R Model, job resources, such as CSS, are associated with work engagement and can buffer the detrimental effects of high job demands on exhaustion, thus reducing the risk of burnout. Furthermore, according to the Conservation of Resources Theory (COR; Hobfoll, 1989, 2001), CSS could also be considered a resource, defined as “objects, personal characteristics, conditions or energies that are valued in their own right or that are valued because they act as conduits to the achievement or protection of valued resources” (Hobfoll, 2001, p. 339). Psychological stress or burnout could occur when resources are threatened, lost, or insufficiently replenished following resource investment. From this perspective, CSS may help prevent burnout by enabling individuals to preserve and build their resource base, thereby reducing resource depletion and strengthening their capacity to cope with stress (Hobfoll, 2001). As for Self-Determination Theory (SDT; Deci and Ryan, 1985), positive work relationships, including those with colleagues, contribute to the satisfaction of the three basic psychological needs of autonomy, competence, and relatedness, thereby increasing autonomous motivation and reducing the risk of burnout. Although all three models underscore the protective role of CSS, they differ in the mechanisms that may explain its relationship with burnout: the JD–R Model emphasizes its buffering role, COR Theory highlights its contribution in preserving and accumulating resources, whereas SDT explains its effect on burnout through the satisfaction of basic psychological needs and autonomous motivation.

From an empirical perspective, several studies have also found a significant association between CSS and burnout, where a high level of support from colleagues is usually associated with a lower level of burnout (Avanzi et al., 2018; Ho, 2016; Kilroy et al., 2022). On the other hand, some studies have found no direct relationship between CSS and burnout (Javaid et al., 2023) or have found that CSS can increase the risk of burnout (Nielsen et al., 2023). These contradictory results may be due to inconsistency in the definition and measurement of social support and burnout. Indeed, there is no universally accepted measure of social support at work (Jolly et al., 2021). Studies have usually used various scales that are often generic and do not distinguish between the specific sources and functions of social support. Meanwhile, burnout has also been the subject of controversy in the scientific community, mainly regarding its definition, dimensions, measurement and classification as a mental disorder (Heinemann and Heinemann, 2017; Nadon et al., 2022; Tavella et al., 2020).

Given the growing and sometimes contradictory body of scientific literature examining the association between CSS and burnout, it seems necessary to take a step back and analyze the work accomplished so far. This article therefore aims to conduct a scoping review that examines the extent, range and nature of the scientific literature on the relationship between CSS and burnout.

## 2 Materials and methods

In accordance with Arksey and O’Malley’s (2005) methodological framework, the scoping review was carried out in the following five stages: (1) identifying the research question; (2) identifying relevant studies; (3) selecting the studies; (4) charting the data; and (5) collating, summarizing and reporting the results. The present scoping review also conformed to the PRISMA Extension for Scoping Reviews (PRISMA-ScR) guidelines (Tricco et al., 2018).

### 2.1 Identifying the research question

This study aimed to answer the following research question: What are the characteristics of the quantitative literature examining the relationship between social support from colleagues and burnout? Accordingly, the review sought to identify studies conducted among workers (population) and quantitatively examining the relationship between different types of social support (e.g., emotional, instrumental) from colleagues and burnout (concepts), without restricting the search to a specific work context, occupation, or geographic location (context).

The main goal of this review was to map the extent, range and nature of the scientific literature on this subject. Given the heterogeneity in the definitions and measurement of workplace social support (Jolly et al., 2021), the review also examined how colleagues’ social support and burnout were operationalized and measured across studies. Finally, in light of the increasingly diverse composition of today’s workforce (Roberson, 2019), the review assessed the extent to which the identified studies included participants from diverse or minority groups (e.g., gender, ethnic, or sexual minority groups).

### 2.2 Identifying relevant studies

In order to answer the research question, a first extraction of the relevant studies was performed in January 2024. A second extraction was performed in February 2025 in order to update the review. After all the article’s authors were consulted and gave their approval, the following search query was used: (“social support” OR (coworker* W/3 support) OR (colleague* W/3 support) OR (team-member* W/3 support)) AND (burnout OR “emotional exhaustion” OR “mental health” OR “psychological health” OR “psychological distress” OR “well-being” OR wellbeing OR wellness) AND (colleague* OR coworker*). Boolean operators varied from one database to another, but the same keywords were used in the nine databases consulted: Scopus, PsycINFO, PsyARTICLES, Business Source Complete, ABI/Inform collection, Psychology and Behavioral Sciences Collection, Érudit, Cairn and OpenEdition (see Supplementary material 1 for the full search strategy in each database). As shown in Figure 1, the extractions resulted in a total of 2,647 articles. After 721 duplicates were removed, a total of 1,926 references were retained for the screening stage.

**Figure 1:**
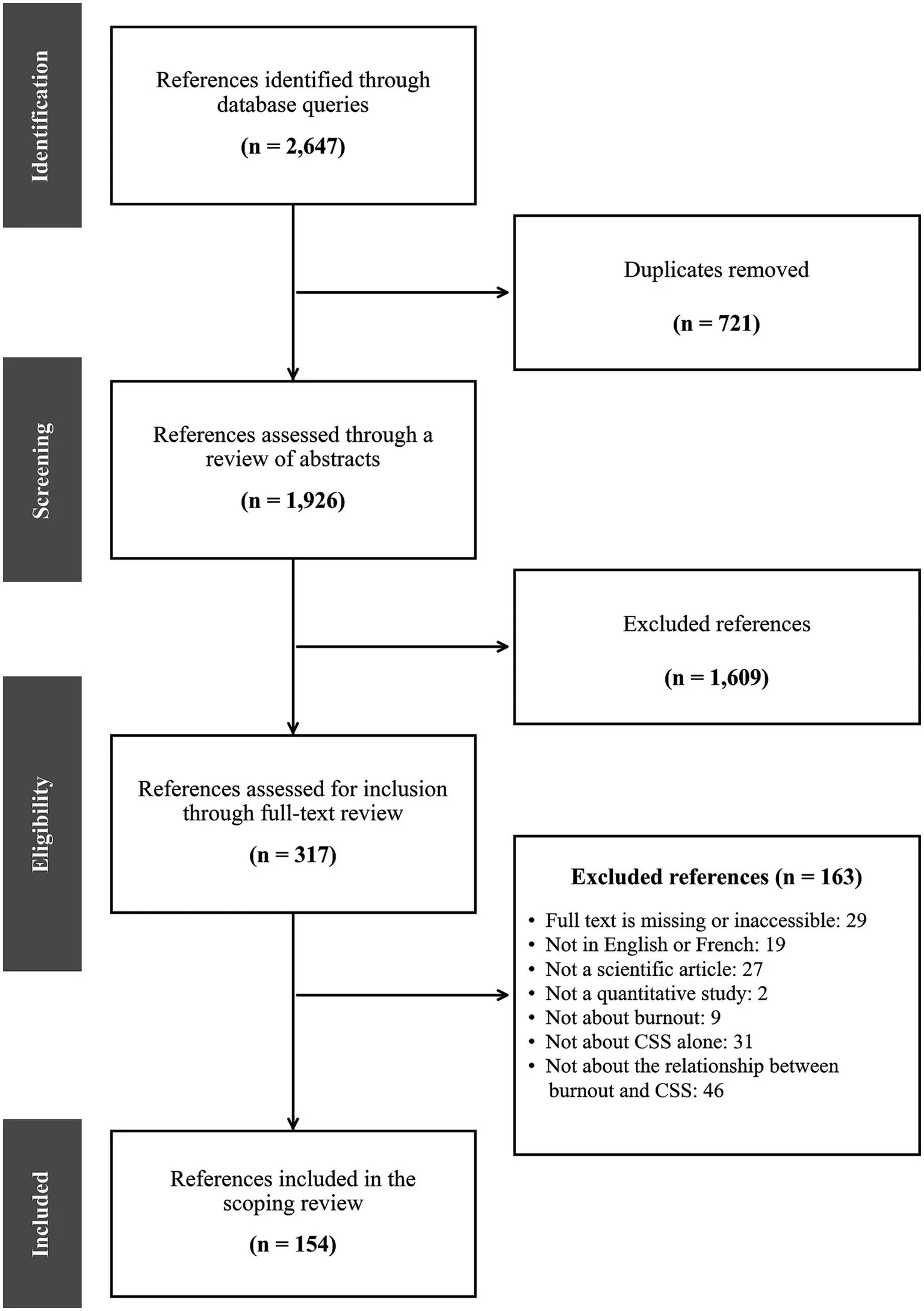
Flow diagram of articles identified and excluded according to PRISMA guidelines. CSS = Colleague social support.

### 2.3 Selecting the studies

The references retained were first screened by reading the abstracts and applying the inclusion criteria presented in Table 1. First (1), the abstract had to be accessible. Then, the full articles had to: (2) be accessible; (3) be written in English or French; (4) be published in a peer-reviewed journal; (5) focus on colleagues’ social support and burnout; and (6) quantitatively examine the relationship between social support from colleagues and burnout.

**Table 1:** Inclusion criteria.

Inclusion criteria
Abstract is available.
Full publication is available.
Full publication is written in English or French.
Study is published in a peer-reviewed journal.
Study must be at least partly quantitative.
Quantitative component deals with burnout (must be defined as such) or at least one of its sub-dimensions.
Quantitative component deals with social support from colleagues measured independently of other sources of social support.
Quantitative component deals with the relationship between colleagues’ social support and at least one burnout dimension.

One of the authors (MR) proceeded with the initial screening of titles and abstracts. In the interest of methodological rigor, another author (FLJ) also screened 5% of the abstracts. As the inter-rater agreement was good (*κ* = 0.92), the remaining abstracts were independently assessed by a single author (MR). Based on titles and abstracts, a total of 1,609 references were excluded from the study because they did not meet the inclusion criteria (see Table 1). Two of the authors (MR and ÉL) proceeded with a full-text screening of the 317 remaining references. As a result of this process, 154 articles were included in the present scoping review (see Figure 1).

### 2.4 Charting the data

The pertinent information extracted from the 154 references included in this scoping review were organized in a data charting form (using Microsoft Excel; Arksey and O’Malley, 2005). The following information was extracted: author(s), year of publication, journal, language of publication, study design, sample characteristics (number of participants, mean age, gender, job type, country), how CSS was conceptualized, CSS measure (e.g., questionnaire used, number of items), how burnout was conceptualized, burnout measure, the role of CSS regarding burnout (e.g., predictor), the theoretical model used, and whether or not the relationship between CSS and burnout was the article’s main focus. In addition, in accordance with current Equity, Diversity and Inclusion (EDI) practices, information about whether the article focusses on a source of diversity (e.g., ethnicity, sexual orientation), mental health issues or sex and gender analysis, was extracted. The Excel table was completed by two separate reviewers (MR and ÉL) and is available as Supplementary material 2.

### 2.5 Collating, summarizing, and reporting the results

The authors summarized and reported the results by organizing them by theme in the Excel table. Several decisions had to be made to summarize the reporting of findings. For example, since numerous job titles were reported across the included studies, one author (MR) classified them into broad job sectors, based on the North American Industry Classification System (NAICS) (Statistics Canada, 2022). In addition, since numerous colleague support questionnaires were identified, questionnaires with insufficient frequency (≤3) were grouped under the “Other” label. Furthermore, as suggested by Arksey and O’Malley (2005), two experts in social support and psychological health at work (SM and FLJ) were consulted at each stage of the scoping review, especially to highlight the most relevant elements to report in the results.

## 3 Results

### 3.1 Publication year

As presented in Figure 2, the number of publications examining the relationship between CSS and burnout has increased considerably over the years, highlighting the growing scientific interest in this topic. Most of the studies (*n* = 99, 64.3%) were published between 2016 and 2025. To our knowledge, the earliest available quantitative study that investigated the relationship between CSS and burnout was by Schwab et al. (1986), which studied the sources and consequences of burnout in a sample of teachers.

**Figure 2:**
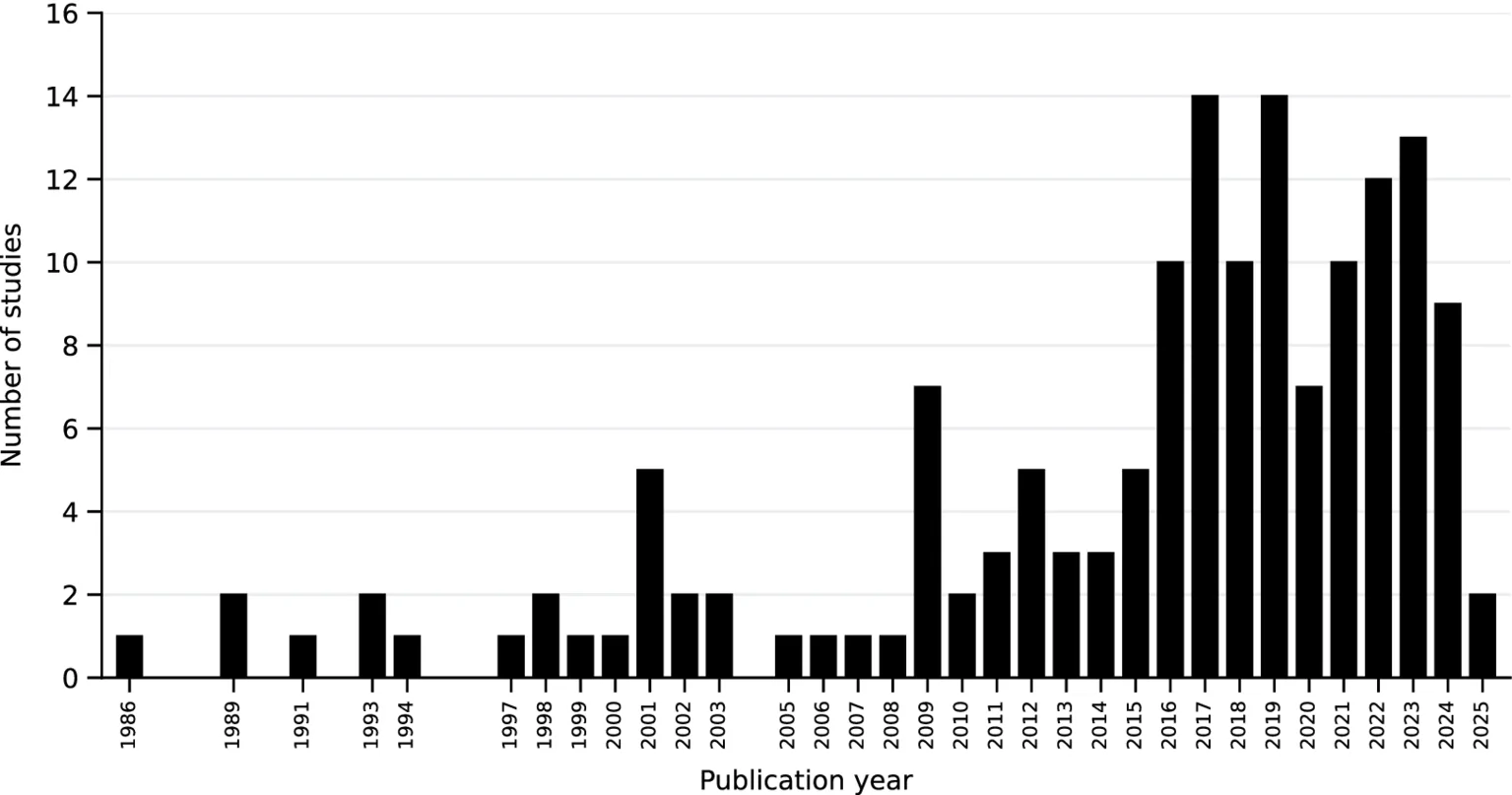
Publication year of the included studies (*n* = 154). Note that the data extraction was completed in February 2025.

### 3.2 General information on included studies

The vast majority (*n* = 153, 99.4%) of the selected articles were written in English, with only one being written in French (Wodociag et al., 2019). Studies were mainly conducted in Europe (*n* = 66, 42.9%), especially the Netherlands (*n* = 15, 9.7%), Italy (*n* = 11, 7.1%) and Norway (*n* = 7, 4.5%), as well as in North America (*n* = 39, 25.3%), especially in the United States (*n* = 30, 19.5%). Very few, if any, studies were conducted in Africa (*n* = 2, 1.3%) or South America (*n* = 0, 0%). Out of the 154 publications, the smallest sample size was 36, while the largest was 34,771 participants. Additionally, most of the included studies were cross-sectional (*n* = 136, 88.3%), limiting the ability to draw conclusions regarding the directionality of the relationship between CSS and burnout.

### 3.3 Sociodemographic characteristics

Table 2 presents the sociodemographic characteristics of the participants in the included studies. Most studies had participants aged between 30 and 49 years old (*n* = 93, 60.4%). Among the samples examined, the lowest mean age was 20.94 years old, while the highest mean age was 54.60 years old. Moreover, most studies recruited participants from the “Health and social services” (*n* = 74, 48.1%) and “Education” (*n* = 26, 16.9%) occupational sectors. Within these two sectors, each had an occupation that stood out: 27.0% (*n* = 20; e.g., Kilroy et al., 2022) of the participants in the “Health and social services” occupational sector were working as a “nurse” and 61.5% (*n* = 16; e.g., Ho, 2016) of the individuals in the “Education” occupational sector were working as a “teacher.” Most studies (*n* = 110, 71.4%) were composed of a larger proportion of women, with nine studies exclusively composed of women. Studies with more than 75% of men (*n* = 18, 11.7%) were predominantly conducted in the “Industry & technology” and the “Health and social services” sectors. Thus, studies examining the relationship between CSS and burnout appear to have been conducted primarily in female-dominated occupational sectors. Few studies have investigated how this relationship may unfold across a broader range of occupational contexts, particularly in primary and secondary sectors (e.g., construction, manufacturing) and among male workers, highlighting a potential gap in the literature.

**Table 2:** Sociodemographic characteristics of the included studies (*n* = 154).

Sociodemographic characteristics	*n* (%)^*^
Sample size	
<200	45 (29.2)
200–399	49 (31.8)
400–999	45 (29.2)
≥1,000	25 (16.2)
Mean age	
<30	10 (6.5)
30–39	45 (29.2)
40–49	48 (31.2)
≥50	2 (1.3)
N/A	42 (27.3)
Proportion of men	
0–24%	56 (36.4)
25–49%	54 (35.1)
50–74%	28 (18.2)
75–100%	18 (11.7)
N/A	7 (4.5)
Recruitment continent	
Europe	66 (42.9)
North America	39 (25.3)
Asia	35 (22.7)
Oceania	6 (3.9)
Africa	2 (1.3)
South America	0 (0)
N/A	6 (3.9)
Occupational sector	
Health and social services	74 (48.1)
Education	26 (16.9)
Industry and technology	11 (7.1)
Security	10 (6.5)
Hospitality and customer service	8 (5.2)
Finance and business	4 (2.6)
Other sectors	12 (7.9)
N/A	15 (9.7)

^*^Cumulative percentages may exceed 100% because some articles may include multiple elements of the same variable.

### 3.4 Colleagues’ social support (CSS)

Table 3 presents how CSS was conceptualized and measured in the included studies. Out of the 154 publications, most of them studied emotional (*n* = 25, 16.2%) and instrumental (*n* = 20, 13.0%) support, but the vast majority (*n* = 125, 81.2%) did not specify the type of social support under investigation. This represents a significant conceptual gap, as different types of social support serve distinct functions. For example, emotional support (e.g., listening, empathy) differs substantially from instrumental support (e.g., practical assistance with completing a task) or appraisal support (e.g., feedback on performance). This distinction is particularly important given that the effectiveness of social support may depend on the extent to which the support provided matches employees’ needs in stressful situations (Viswesvaran et al., 1999). Consequently, testing theoretical models or comparing findings across studies that examine CSS as a general construct or that only focus on specific functions of social support remains challenging, as these approaches represent distinct concepts with different theoretical foundations.

**Table 3:** Colleagues’ social support characteristics examined in the included studies (*n* = 154).

Colleagues’ social support characteristics	*n* (%)^*^
Type of colleagues’ social support studied	
Emotional	25 (16.2)
Instrumental	20 (13.0)
Informational	7 (4.5)
Appraisal	6 (3.9)
N/A	125 (81.2)
Colleagues’ social support questionnaire used	
Job Content Questionnaire (JCQ)	22 (14.3)
Copenhagen Psychosocial Questionnaire (COPSOQ)	9 (5.8)
Questionnaire on the Experience and Evaluation of Work (QEEW)	6 (3.9)
Health and Safety Executive Management Standards Indicator Tool (HSEMSIT)	6 (3.9)
Leiden Quality of Work Questionnaire (LQWQ)	4 (2.6)
Survey of Perceived Organizational Support (SPOS)	4 (2.6)
Other	34 (22.1)
N/A	68 (44.2)
Standardization level	
Validated	69 (44.8)
Modified	28 (18.2)
Custom-made	29 (18.8)
N/A	28 (18.2)
Number of items used to measure CSS	
≤3	45 (29.2)
4–6	66 (42.9)
7–9	12 (7.8)
≥10	10 (6.5)
N/A	22 (14.3)

^*^Cumulative percentages may exceed 100% because some articles may include multiple elements of the same variable.

From a methodological standpoint, many studies (*n* = 68, 44.2%) did not mention a name for the questionnaire they were using, making it difficult to fully understand how they operationalized CSS. Among the studies that specified the name of their questionnaire, the most frequently cited were the Job Content Questionnaire (JCQ, *n* = 22, 14.3%), followed by the Copenhagen Psychosocial Questionnaire (COPSOQ, *n* = 9, 5.8%), the Questionnaire on the Experience and Evaluation of Work (QEEW, *n* = 6, 3.9%), the Health and Safety Executive Management Standards Indicator Tool (HSEMSIT, *n* = 6, 3.9%), the Leiden Quality of Work Questionnaire (LQWQ, *n* = 4, 2.6%) and the Survey of Perceived Organizational Support (SPOS, *n* = 4, 2.6%). In addition, 34 studies (22.1%) used a questionnaire that was mentioned less than 4 times across the scoping review, for a total of 28 different “other” questionnaires. Examples of questionnaires used less than 4 times to measure CSS include the Social Support Rating Scale (Ma et al., 2020), the General Nordic Questionnaire (Nielsen et al., 2023), and the Physician Worklife Survey (Starmer et al., 2016). Of all the questionnaires, 29 were custom-made, while 28 were modified version of a validated questionnaire used to measure CSS. For example, Kilroy et al. (2022) modified the SPOS by using 3 of the 36 items and by replacing the concept of “organization” with “colleague.” Most of the included studies (*n* = 111, 72.1%) used less than 7 items to measure CSS, with 9 of them using only one item.

Several shortcomings can be identified regarding the measurement of CSS. Although some items included in the listed questionnaires may refer to different functions of social support (e.g., instrumental support: “People I work with are helpful in getting the job done,” LQWQ; emotional support: “My colleagues are willing to listen to my work-related problems,” HSEMSIT), studies typically rely on a global CSS score for analyses and do not examine whether specific functions have distinct associations with burnout. Furthermore, some questionnaires, such as the QEEW, appear to capture the perceived quality of social relationships with colleagues (e.g., “Do you get on well with your colleagues?”) rather than the specific support provided by colleagues. Additionally, while most questionnaires use agreement scales (e.g., from strongly disagree to strongly agree), others—such as the COPSOQ—use frequency scales (e.g., “How often do you get help and support from your colleagues, if needed?”), thereby assessing another aspect of social support—its perceived frequency. In sum, the wide variety of questionnaires used to measure CSS raises questions regarding whether these measures capture the same underlying construct and limits comparability across findings. Furthermore, studies relying on a global score of CSS, without distinguishing among the four functions of social support described by House (1981), can contribute to create a disconnect between existing theoretical frameworks and the operationalization of CSS.

### 3.5 Burnout

Table 4 presents how burnout was conceptualized and measured in the included studies. In contrast to CSS, most publications (*n* = 144, 93.5%) used a validated questionnaire to measure burnout, with the most widely used one being the Maslach Burnout Inventory (MBI, *n* = 101, 65.6%). In accordance with Maslach’s conceptualization of burnout, the majority of studies investigated one of the three main dimensions of burnout separately: emotional exhaustion (*n* = 99, 64.3%), depersonalization, also referred to as cynicism or disengagement (*n* = 60, 39.0%), and reduced professional accomplishment (*n* = 41, 26.6%). Very few studies (*n* = 4, 2.6%) explored more than 3 dimensions of burnout, while some studies investigated burnout as a general concept, without specifying a particular dimension (*n* = 34, 22.1%).

**Table 4:** Burnout characteristics of the included studies (*n* = 154).

Burnout characteristics	*n* (%)^*^
Burnout dimension studied	
Exhaustion or emotional exhaustion	99 (64.3)
Depersonalization, cynicism or disengagement	60 (39.0)
Reduced professional efficacy/accomplishment	41 (26.6)
Other	12 (7.8)
N/A	34 (22.1)
Number of burnout dimensions studied	
0	34 (22.1)
1	46 (29.9)
2	23 (14.9)
3	47 (30.5)
4	4 (2.6)
Burnout questionnaire used	
Maslach Burnout Inventory (MBI)	101 (65.6)
Copenhagen Burnout Inventory (CBI)	12 (7.8)
Copenhagen Psychosocial Questionnaire (COPSOQ)	9 (5.8)
Burnout Assessment Tool (BAT)	5 (3.2)
Oldenburg Burnout Inventory (OLBI)	5 (3.2)
Shirom-Melamed Burnout Measure (SMBM)	2 (1.3)
Professional Quality of Life Scale (ProQOL)	3 (1.9)
Other or N/A	17 (11.0)

^*^Cumulative percentages may exceed 100% because some articles may include multiple elements of the same variable.

Although the conceptualization and measurement of burnout appeared to be more consistent than those of CSS across included studies, questions remain as to whether measures focusing solely on one dimension (e.g., emotional exhaustion) adequately capture the construct of burnout. Furthermore, although the Maslach Burnout Inventory (MBI) remains the most widely used measure, other questionnaires, such as the Copenhagen Burnout Inventory (CBI), the Copenhagen Psychosocial Questionnaire (COPSOQ), the Burnout Assessment Tool (BAT), and the Oldenburg Burnout Inventory (OLBI), are also used. However, these instruments are based on different theoretical conceptualizations of burnout, which may contribute to heterogeneity in future research. For example, the OLBI conceptualizes burnout through two dimensions: exhaustion and disengagement, whereas the CBI distinguishes among personal burnout, work-related burnout, and client-related burnout. These approaches may therefore capture distinct manifestations of burnout and limit comparability across studies.

### 3.6 The relationship between CSS and burnout

The relationship between burnout and social support from colleagues was the main focus of only 56 (36.4%) of the 154 included articles. All of these, however, investigated CSS as a predictor of burnout, although CSS was most often considered alongside other potential predictors. To explain the relationship between CSS and burnout, several studies (*n* = 48, 31.2%) used the Job Demand-Resources Model (JD-R) as their main theoretical model, conceptualizing CSS as a job resource that can foster engagement and buffer the impact of job demands on exhaustion. Other theoretical models used in the studies included the Conservation of Resources Theory (COR, *n* = 28, 18.2%), which emphasizes the role of CSS in protecting employees from resource loss, and the Self-Determination Theory (SDT, *n* = 4, 2.6%), which highlights the contribution of supportive relationships to the satisfaction of employees’ basic psychological needs, particularly the need for relatedness.

### 3.7 Focus on diversity

Of the 154 articles included in the scoping review, only 12 (7.8%) discussed an aspect of diversity. Eight (5.2%) studies focused on women, while only one (0.65%) study looked at older workers, two (1.3%) explored the experiences of Chinese expatriates and one (0.65%) investigated the experiences of African American individuals. Additionally, only eight (5.2%) of the 154 included studies carried out gender-based analyses, while none of them conducted sex- or gender-sex based analyses. Moreover, only four (2.6%) articles focused on CSS and burnout in interaction with another mental health issue, namely post-traumatic stress disorder or exposure to traumatic events (Bell et al., 2019; Boerner et al., 2017; Kleineidam and Fischbach, 2024) and chronic mental and physical illnesses (Boelhouwer et al., 2020). Studies examining the relationship between CSS and burnout have therefore been conducted primarily among relatively homogeneous populations, with limited representation of diverse or minority groups. As a result, little is known about whether the relationship between CSS and burnout differs across these groups.

## 4 Discussion

The purpose of this study was to map the extent, range and nature of the quantitative scientific literature examining the relationship between CSS and burnout. While the first studies identified dated from the 1980s and were thus in line with the initial emergence of the concept of burnout, most studies were published between 2016 and 2025, showing a growing interest in the subject over the past decade. This could be explained by the fact that psychological health problems affect a great number of workers and their deleterious impact on the professional and personal spheres is being increasingly recognized (Malik et al., 2023; World Health Organization, 2024).

Although many studies were identified in the present review, the specific association between CSS and burnout was the primary focus of only one third of them. Most studies examined CSS alongside a range of other predictors, highlighting the need for future research to explore the specific relationship between CSS and burnout in greater depth. In addition, although different functions of social support have been recognized in the scientific literature (e.g., emotional, instrumental, informational, appraisal; House, 1981), CSS was mainly operationalized as a global construct in the included studies. Indeed, most studies did not specify the type (function) of CSS they were measuring, and nearly a third of them used three items or fewer to measure CSS, limiting their ability to capture its multidimensional nature. A large number of questionnaires, varying from one study to another, were also used to measure CSS. This may be explained by the fact that there is currently no gold-standard measure for CSS (Jolly et al., 2021). Furthermore, most existing questionnaires are incomplete and fail to measure all possible sources or functions of social support (Jolly et al., 2021). As a result, researchers must choose among the various incomplete scales that already exist and sometimes modify or create new ones in order to achieve their objectives. This variability in the operationalization and measurement of CSS raises important questions regarding its construct validity. The lack of standardization across studies also limits the comparison of findings and may hinder the development of new knowledge.

Future research would benefit from the development and validation of a theoretically grounded comprehensive measure of social support at work that distinguishes the sources of support and thus isolates the effect of CSS. In addition, such a measure should provide specific information about the distinct functions of social support recognized in the literature, such as emotional support, instrumental support, informational support and appraisal support (House, 1981). A nuanced conceptualization and measurement of social support could facilitate the development and refinement of targeted interventions aimed at strengthening different type of social support and preventing burnout.

Less variability was found among the selected studies regarding burnout: the MBI remained the most widely used questionnaire, although there is a trend toward the use of other questionnaires like the CBI or the COPSOQ. However, with both MBI and the other questionnaires, studies varied in terms of which of the three dimensions of burnout they considered. The majority of the selected studies used a global score or only considered one or two dimensions of burnout, demonstrating a partial approach to the phenomenon that potentially prevents us from understanding it in its full complexity. This diversity of application, often linked to the context of the study or the objectives of the researchers, makes comparisons between results difficult. Moreover, although recognized as a valid measure of this concept, the use of self-reported measures such as the MBI to assess burnout may result in common variance or social desirability biases. Future studies may benefit from turning to more objective measures of burnout. Recent studies indicate that heart rate variability (Wekenborg et al., 2019; Wekenborg et al., 2022) or cortisol levels (Marchand et al., 2014; Zuccarella-Hackl et al., 2024) may be potential indicators for burnout and could be used in future studies investigating the association between CSS and burnout.

Most studies of the present review were also cross-sectional and considered CSS as an antecedent of burnout, but never the other way around. Consistent with theoretical frameworks such as the Job Demands–Resources Model (JD-R; Bakker and Demerouti, 2007), Conservation of Resources Theory (COR; Hobfoll, 1989, 2001), and Self-Determination Theory (SDT; Deci and Ryan, 1985), CSS was conceptualized as a resource that helps individuals cope with job demands, better manage work-related stress, and satisfy their basic psychological needs. However, considering the possibility that burnout could also alter the quantity and quality of CSS, the design of these studies serves as an obstacle to fully understanding the complexity of the relationship between CSS and burnout. In this regard, studies conducted in different contexts have identified reciprocal, evolving relationships between social support and various indicators of psychological distress (Debnar et al., 2024; Robitaille et al., 2012; Sconfienza et al., 2019). From a theoretical perspective, the JD-R model (Bakker and Demerouti, 2007) also posits a feedback loop between burnout and job demands and resources, such as social support, which remains underexamined in the current literature focusing on CSS and burnout. More longitudinal studies are thus needed to properly document the direction of the relationship between CSS and burnout, as well as its evolution over time. Experimental designs aiming at manipulating or improving CSS could also be useful in verifying its causal relationship with burnout.

In another vein, the results of this scoping review show that the relationship between CSS and burnout was examined in quite homogeneous samples. First, most studies thus far have been conducted with workers aged between 30 and 49 years old. This demonstrates the lack of studies including older or younger people, for whom social support at work might have different significance (Krause and Rainville, 2020; Kröner and Mueller, 2022). Also, most studies were conducted among women in the “Health and social services” and “Education” sectors. This could be explained by the fact that the concept of burnout has been first and foremost examined in these occupational contexts (Schaufeli and Buunk, 2003). However, it is now recognized that burnout can affect workers in any sector (Edú-Valsania et al., 2022). Future research should be conducted in other, less represented work sectors such as “Construction,” “Security,” “Hospitality and customer service”, and “Finance and business”. In addition, most of the included studies were conducted in Europe and North America, while few, if any, took place in Africa and South America. This imbalance can be explained by factors such as underfunding of research or language barriers in some countries or continents (Ger et al., 2024; Quiroga-Garza et al., 2022). It is essential that future studies be conducted in these continents to promote equity in research, to enrich global understanding of the phenomenon and to avoid conclusions limited to Western contexts. Finally, very few studies have taken aspects of diversity into account (i.e., gender, ethnicity). Indeed, the majority of the included studies have examined the association between CSS and burnout globally, without considering individual characteristics that could influence it. Yet social support may vary according to individual characteristics such as gender, age and ethnicity (Ritchie-Dunham et al., 2025).

This study contributes to characterizing154 studies examining the association between CSS and burnout. Such mapping has not been done in this field which is always evolving and in need of synthesis and future direction. Despite its contribution to the understanding and contextualizing of future research focusing on CSS and burnout, this study has some limitations. First, only French or English publications were considered, and relevant studies written in other languages could not be included. Second, although our search strategy was designed to maximize sensitivity while maintaining specificity, the selection of keywords may have influenced the studies identified. In particular, we focused on terms referring specifically to social support from colleagues, and did not include broader or related concepts (e.g., *peer support*, *team support*, or *workplace support*), which may have resulted in some relevant studies being overlooked. Third, given the large amount of existing literature on social support and burnout, only quantitative studies were considered, thus eliminating all qualitative publications on the subject. Future reviews would benefit from the inclusion of qualitative studies in order to inspire future research aiming at the in-depth exploration of the specific dynamics of this relationship. Finally, although burnout is one of the most commonly used variables to represent mental health at work, broader indicators of psychological health should be considered in further research (e.g., engagement, psychological distress, anxiety and depression).

## 5 Conclusion

This scoping review represents a first attempt to map the existing quantitative literature on the relationship between CSS and burnout, encompassing more than 150 studies. The findings reveal a fragmented landscape, partly due to the to substantial variability in the conceptualisation and measurement of these constructs across studies. Much work remains to be done to provide a clearer and more accurate portrait of the association between CSS and burnout. A more nuanced conceptualization of CSS, burnout and their interrelationship could pave the way for applied research, intervention and, ultimately, improvements in workplace mental health. Future research should prioritize longitudinal designs and employ standardized, multidimensional measures of social support to enhance comparability and construct validity. Moreover, studies involving more heterogeneous populations are essential to better reflect the diversity of today’s workforce. Overall, this scoping review will help researchers better conceptualize future studies in hope of a more comprehensive evidence base of the relationship between CSS and burnout.

## Data Availability

The original contributions presented in the study are included in the article/Supplementary material, further inquiries can be directed to the corresponding author.
