# Supplementary material for: The relationship between colleagues’ social support and burnout: a scoping review of quantitative research
Source: Front Psychol. 2026 Oct 6;17:1858058. doi: 10.3389/fpsyg.2026.1858058 (PMC13680209; doi:10.3389/fpsyg.2026.1858058)
Supplement: Supplementary file 1 [file Table_1.DOCX]

**Supplementary Material: Full Database-Specific Search Strategies**

This supplementary material documents the complete database-specific search strategies used in the scoping review "The relationship between colleagues’ social support and burnout: A scoping review of quantitative research". Literature searches were conducted in nine academic databases across two extraction rounds: an initial extraction in January 2024 and a search update in February 2025. Exact query syntax, database search fields, proximity operators, language filters, and the number of records retrieved/imported are detailed below.

**Table S1. Initial Database Search Strategies (January 2024)**

| **Database** | **Search Fields** | **Exact Search Query** | **Hits Imported** |
| --- | --- | --- | --- |
| Scopus (Elsevier) | Title, Abstract, Keywords (TITLE-ABS-KEY) | TITLE-ABS-KEY ( ( "social support" OR coworker* W/3 support OR colleague* W/3 support OR team-member* W/3 support ) AND ( "mental health" OR "psychological health" OR "psychological distress" OR burnout OR "well-being" OR wellbeing OR wellness OR "emotional exhaustion" ) AND ( colleague* OR coworker* ) ) | 1,345 |
| PsycInfo (APA PsycNet) | Title, Keywords, Abstract | (("social support" OR (coworker* NEAR/3 support) OR (colleague* NEAR/3 support) OR (team-member* NEAR/3 support)) AND ("mental health" OR "psychological health" OR "psychological distress" OR burnout OR "well-being" OR wellbeing OR wellness OR "emotional exhaustion") AND (colleague* OR coworker*)) | 554 |
| PsycArticles (APA PsycNet) | Title, Keywords, Abstract | (("social support" OR (coworker* NEAR/3 support) OR (colleague* NEAR/3 support) OR (team-member* NEAR/3 support)) AND ("mental health" OR "psychological health" OR "psychological distress" OR burnout OR "well-being" OR wellbeing OR wellness OR "emotional exhaustion") AND (colleague* OR coworker*)) | 38 |
| Business Source Complete (EBSCOhost) | Title, Author Keywords, Abstract | (("social support" OR (coworker* N3 support) OR (colleague* N3 support) OR (team-member* N3 support)) AND ("mental health" OR "psychological health" OR "psychological distress" OR burnout OR "well-being" OR wellbeing OR wellness OR "emotional exhaustion") AND (colleague* OR coworker*)) | 139 |
| ABI/Inform Collection (ProQuest) | Title, Abstract | (("social support" OR (coworker* NEAR/3 support) OR (colleague* NEAR/3 support) OR (team-member* NEAR/3 support)) AND ("mental health" OR "psychological health" OR "psychological distress" OR burnout OR "well-being" OR wellbeing OR wellness OR "emotional exhaustion") AND (colleague* OR coworker*)) | 173 |
| Psychology and Behavioral Sciences Collection (EBSCOhost) | Title, Abstract | (("social support" OR (coworker* N3 support) OR (colleague* N3 support) OR (team-member* N3 support)) AND ("mental health" OR "psychological health" OR "psychological distress" OR burnout OR "well-being" OR wellbeing OR wellness OR "emotional exhaustion") AND (colleague* OR coworker*)) | 111 |
| Érudit (French) | Title, Abstract, Keywords | (("soutien social" OU "soutien des collègues" OU "soutien des membres de l’équipe") ET ("santé mentale" OU "santé psychologique" OU "détresse psychologique" OU "épuisement professionnel" OU "bien-être" OU "épuisement émotionnel") ET (collègue*)) | 6 |
| Cairn.info (French) | Title, Abstract | (("soutien social" OU "soutien des collègues" OU "soutien des membres de l’équipe") ET ("santé mentale" OU "santé psychologique" OU "détresse psychologique" OU "épuisement professionnel" OU "bien-être" OU "épuisement émotionnel") ET (collègue*)) | 3 |
| OpenEdition (French) | Title, Abstract | (("soutien social" OR "soutien des collègues" OR "soutien des membres de l’équipe") AND ("santé mentale" OR "santé psychologique" OR "détresse psychologique" OR "épuisement professionnel" OR "bien-être" OR "épuisement émotionnel") AND (collègues)) | 0 |

**Table S2. Updated Database Search Strategies (February 2025)**

| **Database** | **Search Fields & Restrictions** | **Exact Search Query** | **Hits Imported** |
| --- | --- | --- | --- |
| Scopus (Elsevier) | TITLE-ABS-KEY & PUBYEAR > 2023 AND PUBYEAR < 2026 | TITLE-ABS-KEY ( ( "social support" OR coworker* W/3 support OR colleague* W/3 support OR team-member* W/3 support ) AND ( "mental health" OR "psychological health" OR "psychological distress" OR burnout OR "well-being" OR wellbeing OR wellness OR "emotional exhaustion" ) AND ( colleague* OR coworker* ) ) AND PUBYEAR > 2023 AND PUBYEAR < 2026 | 120 |
| PsycInfo (APA PsycNet) | Title, Keywords, Abstract (2024-2025) | (("social support" OR (coworker* NEAR/3 support) OR (colleague* NEAR/3 support) OR (team-member* NEAR/3 support)) AND ("mental health" OR "psychological health" OR "psychological distress" OR burnout OR "well-being" OR wellbeing OR wellness OR "emotional exhaustion") AND (colleague* OR coworker*)) | 70 |
| PsycArticles (APA PsycNet) | Title, Keywords, Abstract (2024-2025) | (("social support" OR (coworker* NEAR/3 support) OR (colleague* NEAR/3 support) OR (team-member* NEAR/3 support)) AND ("mental health" OR "psychological health" OR "psychological distress" OR burnout OR "well-being" OR wellbeing OR wellness OR "emotional exhaustion") AND (colleague* OR coworker*)) | 6 |
| Business Source Complete (EBSCOhost) | Title, Author Keywords, Abstract (2024-2025) | (("social support" OR (coworker* N3 support) OR (colleague* N3 support) OR (team-member* N3 support)) AND ("mental health" OR "psychological health" OR "psychological distress" OR burnout OR "well-being" OR wellbeing OR wellness OR "emotional exhaustion") AND (colleague* OR coworker*)) | 23 |
| ABI/Inform Collection (ProQuest) | Title, Abstract (2024-2025) | (("social support" OR (coworker* NEAR/3 support) OR (colleague* NEAR/3 support) OR (team-member* NEAR/3 support)) AND ("mental health" OR "psychological health" OR "psychological distress" OR burnout OR "well-being" OR wellbeing OR wellness OR "emotional exhaustion") AND (colleague* OR coworker*)) | 40 |
| Psychology and Behavioral Sciences Collection (EBSCOhost) | Title, Abstract (2024-2025) | (("social support" OR (coworker* N3 support) OR (colleague* N3 support) OR (team-member* N3 support)) AND ("mental health" OR "psychological health" OR "psychological distress" OR burnout OR "well-being" OR wellbeing OR wellness OR "emotional exhaustion") AND (colleague* OR coworker*)) | 11 |
| Érudit (French) | Title, Abstract, Keywords (2024-2025) | (("soutien social" OU "soutien des collègues" OU "soutien des membres de l’équipe") ET ("santé mentale" OU "santé psychologique" OU "détresse psychologique" OU "épuisement professionnel" OU "bien-être" OU "épuisement émotionnel") ET (collègue*)) | 7 |
| Cairn.info (French) | Title, Abstract (2024-2025) | (("soutien social" OU "soutien des collègues" OU "soutien des membres de l’équipe") ET ("santé mentale" OU "santé psychologique" OU "détresse psychologique" OU "épuisement professionnel" OU "bien-être" OU "épuisement émotionnel") ET (collègue*)) | 0 |
| OpenEdition (French) | Title, Abstract (2024-2025) | (("soutien social" OR "soutien des collègues" OR "soutien des membres de l’équipe") AND ("santé mentale" OR "santé psychologique" OR "détresse psychologique" OR "épuisement professionnel" OR "bien-être" OR "épuisement émotionnel") AND (collègues)) | 0 |
